# *Campylobacter jejuni* subdural hygroma infection in a 2-year old boy: case report and a brief literature review

**DOI:** 10.1186/s12879-022-07680-0

**Published:** 2022-08-20

**Authors:** Ivana Valenčak-Ignjatić, Nina Krajcar, Diana Didović, Srđan Roglić, Iva Butić, Marko Jelić, Hrvoje Jednačak, Goran Tešović

**Affiliations:** 1 Pediatric Infectious Diseases Department, University Hospital for Infectious Diseases “Dr. Fran Mihaljević”, Mirogojska 8, 10000 Zagreb, Croatia; 2 School of Medicine, University of Zagreb, Šalata 2, 10000 Zagreb, Croatia; 3 School of Dental Medicine, University of Zagreb, Gundulićeva ulica 5, 10000 Zagreb, Croatia; 4 Department of Neurosurgery, University Hospital Centre Zagreb, Kišpatićeva ulica 12, 10000 Zagreb, Croatia

**Keywords:** *Campylobacter jejuni*, Children, 16S rDNA, Subdural hygroma, Meningitis

## Abstract

**Background:**

*Campylobacter jejuni* is a common cause of acute gastroenteritis, but central nervous system infections are rare manifestations of *Campylobacter* infection. Therefore, *C. jejuni* trauma-related subdural hygroma infection in children is poorly described in the literature.

**Case presentation:**

We described a 2-year old boy with lobar holoprosencephaly presenting with subdural hygroma following head trauma. *C. jejuni* infection was confirmed from a subdural hygroma sample by culture as well as by DNA sequencing of a broad range 16S rDNA PCR product. Cerebrospinal fluid from the ventriculoperitoneal shunt remained sterile. Combined neurosurgical and antimicrobial treatment led to complete recovery. Review of the literature showed that the most common manifestation of *Campylobacter* central nervous system infection is meningitis, mostly in neonates, and subdural hygroma infection was described for only one case.

**Conclusions:**

Subdural hygroma infection caused by *C. jejuni* is a rare clinical condition in children. Molecular methods represent an important tool for the detection of rare or unexpected pathogens. No standard recommendations for antimicrobial treatment of *C. jejuni* subdural space infection in children are available, but meropenem treatment combined with surgery seems to be an effective approach.

## Background

*Campylobacter jejuni* is a major global gastrointestinal pathogen causing enteritis in all age groups of which children under 5 years are commonly infected [1]. According to the European Centre for Disease Prevention and Control *Camplyobacter spp*. has been the most commonly reported gastrointestinal bacterial pathogen since 2005 [2]. Data regarding human campylobacteriosis in Croatia are insufficient and notification of the disease remains manly on physicians reporting clinical cases. Published data from Croatian Institute of Public Health shows relatively consistent number of reported cases in the past 10 years, with notification rate below 49.99 cases per 100,000 [2, 3]. Extraintestinal manifestations of *C. jejuni* infection in children are rare, and include bacteremia, osteomyelitis and meningitis [1, 4–6]. Children with brain abnormalities and previous neurosurgical procedures are at risk for central nervous system (CNS) infections and together with neonates represent the only pediatric population with proven *C. jejuni* CNS infection. Trauma-related subdural hygroma infection has a wide spectrum of clinical manifestations. Symptoms may not be present until weeks after closed head trauma. Since CNS infections are rare manifestations of *C. jejuni* infection, correct and early detection of causative agent is challenging. Isolation of *Campylobacter* in blood and cerebrospinal fluid (CSF) samples requires microaerophilic atmosphere and sometimes enrichment, which is not routinely done for blood and CSF culture [7]. Molecular methods represent another diagnostic option, especially if antimicrobial treatment has been started prior to bacterial sampling. The increasing use of molecular methods for detection of *Campylobacter* is justifiable because of the limitations of the conventional tests. Rapid bacteriological diagnostics is crucial for adequate treatment, especially in CNS infection. The use of 16S rDNA gene sequencing is common for the identification of biochemically unidentified bacteria and unusual strains [8].

We present a rare case of *C. jejuni* subdural hygroma infection in a 2-year-old boy along with detailed clinical course, diagnostic and therapeutic approach, and review available literature on CNS infections caused by *Campylobacter* in children.

## Case presentation

Our patient was born from a twin pregnancy with a lobar holoprosencephaly diagnosed on fetal ultrasound and confirmed on magnetic resonance imaging (MRI) at 27 weeks of gestation. Ventriculoperitoneal (VP) shunt was placed on the 16th day of life to treat consequent hydrocephalus. The patient suffered several VP shunt infections during the early infancy and the last replacement of the shunt system was done at 7 months of age. Surprisingly, his motoric and cognitive development was unremarkable and without delay. Vaccinations were followed up according to the Croatian National Immunization Program, including 13-valent pneumococcal conjugated vaccine. At two years of age he was admitted to the University Hospital for Infectious Diseases “Dr.Fran Mihaljević”, Zagreb, Croatia with a 10-day history of high fever without other clinical signs nor symptoms. Previous examinations at the emergency department on the 1st and 6th day of fever revealed no pathological findings and showed normal complete blood counts with slightly elevated C-reactive protein (CRP) of 26.5 mg/L and unremarkable result of urine sediment. Of relevance from the case history was a report of a closed head trauma without loss of consciousness after falling off a swing two weeks before the fever onset.

On admission to the hospital, his vital signs were normal besides the fever (40 °C), and no pathological findings were evident on neither physical nor neurological examination. His general clinical condition was good. The white blood cell count was 23.4 × 10^9^/L (58.6% neutrophils), CRP and procalcitonin were 383.6 mg/L and 1.29 μg/L, respectively. The erythrocyte sedimentation rate was 110 mm/h. Coagulation tests showed elevated fibrinogen (9.6 g/L) and D-dimers (1.89 mg/L) while renal and liver tests were unremarkable.

### Microbiological diagnosis and treatment

Blood culture sample was drawn into a pediatric bottle from peripheral vein and remained sterile after five days of incubation at 35 ± 1 °C in automated system (BAC/ALERT 3D instrument, BioMerieux, France). One bag urine sample, obtained and cultivated on the blood agar for 24 h at 35 ± 1 °C, remained sterile. Stool sample tested negative for rotavirus and adenovirus antigen. Microbiological analysis also included the common bacterial gastrointestinal pathogens *(Campylobacter* spp., *Salmonella* spp., *Shigella* spp., *Yersinia entereocolitica*). The stool sample after cultivation on the Campylobacter Blood-Free Selective Agar Base (Oxoid, UK) at 42 ± 1 °C for 48 h in microaerophilic condition, on the Yersinia Selective Agar Base (Oxoid, UK) at 29 ± 1 °C for 48 h and Xylose, lysine, deoxycolate agar (Oxoid, UK) at 35 ± 1 °C for 24 h remained negative on tested pathogens. To rule out infection in the VP shunt, a sample from the valve reservoir was obtained percutaneously. Initial CSF microscopy showed a white cell count of 5/3 cmm (monocyte 100%), the protein level of 0.17 g/L, and glucose concentration of 4.2 mmol/L. A CSF sample remained sterile after four days of incubation at 35 ± 1 °C on the blood agar in a humidified 5 to10% CO_2_ atmosphere and in the tryptic soy broth and in room air, respectively. All samples were taken prior to initiation of empirical antimicrobial therapy.

**Fig. 1:**
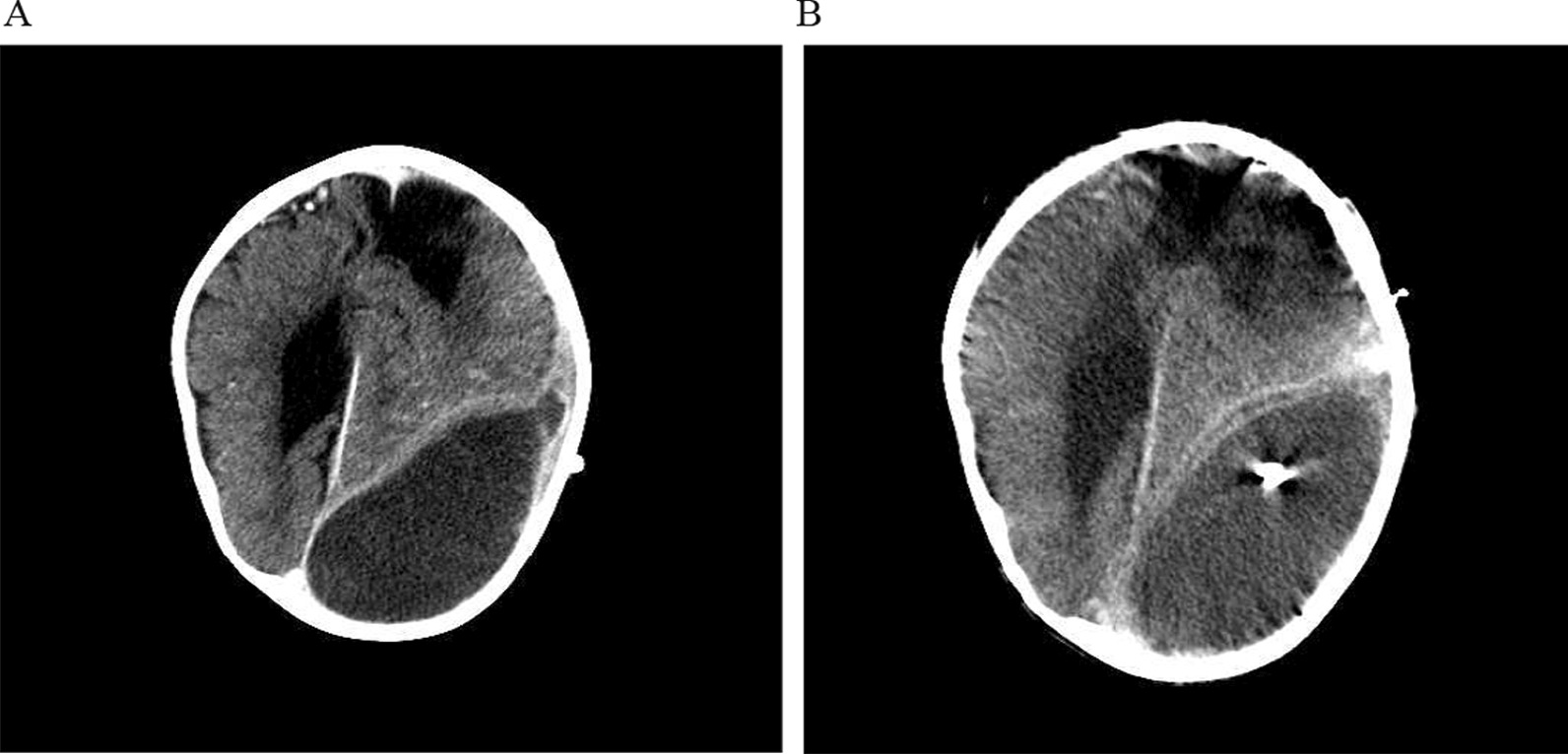
Contrast-enhanced brain computer tomography of the patient showing parietooccipital subdural hygroma on the left side **a** on the 3rd hospitalization day and **b** after insertion of subdural external drainage system

**Fig. 2:**
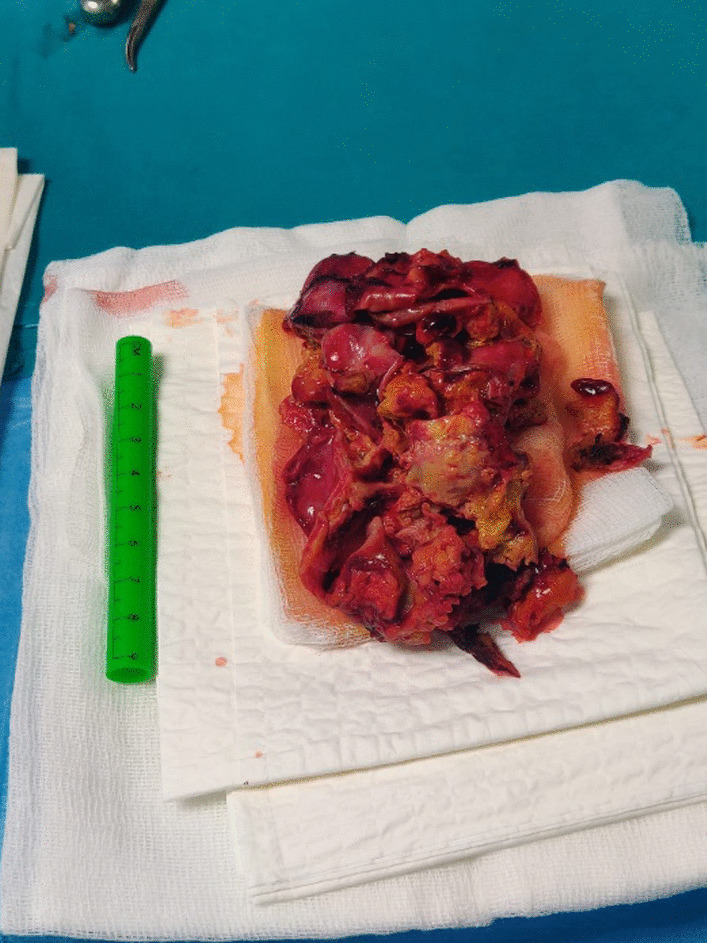
Subdural hygroma/hematoma after surgical removal

Abdominal ultrasound demonstrated a slightly thickened bladder wall and a small amount of free fluid in the abdomen. Empirical therapy with intravenous ceftriaxone was started. However, despite antimicrobial therapy patient continuously had high fever and on the 3rd hospitalization day parents suspected pain in the child’s left ear. Otoscopy revealed no signs of acute inflammation. A contrast-enhanced brain computer tomography (CT) demonstrated parietooccipital subdural hygroma on the left side with signs of inflammation (Fig. 1a). Antimicrobial treatment was switched to vancomycin and meropenem. The patient was transferred to the Department of Neurosurgery and a subdural external drainage (SED) system was inserted. An additional CSF sample was obtained from the SED system and sent to microbiology laboratory for cultivation and PCR testing. Presence of bacterial DNA was detected in the subdural hygroma sample using broad range PCR targeting 16S rDNA with 16sF557 (5’-GACTCCTACGGGAGGCAG-3’) and 16sR1308 (5’-CGCTCGTTGCGGGACTTAAC-3’) primers. DNA sequencing of a PCR product was performed by ABI PRISM 310 Genetic Analyzer using BigDye v1.1 chemistry (ThermoFisher Scientific, USA). Comparison of obtained sequences to NR/NT database using Nucleotide Basic Local Alignment Search Tool (BLASTn) revealed a match to *Campylobacter spp.* (the most significant alignment with accession number: CP040607.1) [9]. The colony growth of *Campylobacter jejuni* was present in the sample of subdural hygroma fluid after 48 h of incubation. Identification was done based on the colony morphology, Gram stain, catalase test, oxidase test and differentiated from *Campylobacter coli* by positive hippurate hydrolysis test. Following the guidelines of European Committee on Antimicrobial Susceptibility Testing the *C. jejuni* isolate was found sensitive to erythromycin, gentamycin, and tetracycline, while resistant to ciprofloxacin [10]. The isolate was susceptible to meropenem (MIC 0.023 mg/L) following the recommendation of Clinical and Laboratory Standards Institute; therefore meropenem monotherapy was continued [11]. Despite drainage system insertion and antimicrobial treatment the fever persisted for nine days. Blood samples were taken on the 2nd and 7th day after the drainage system insertion and showed elevated CRP value (169.8 mg/L and 177.9 mg/L, respectively). Brain CT showed partial regression of hygroma with hyperdense areas, more pronounced dorsally and cranially corresponding to the hemorrhagic content (Fig. 1b). On the 22nd day of illness, the patient underwent another neurosurgical procedure. Craniotomy with total subdural hematoma extirpation was performed, VP shunt was removed and an external ventricular drainage (EVD) system was placed (Fig. 2). Meropenem treatment was continued for a total of 4-weeks and a new VP shunt was inserted.

### Clinical outcome

Two weeks after the termination of the antimicrobial treatment clinical and laboratory evaluation reported no signs of CNS infection as the CSF samples were unremarkable as well as laboratory findings. Eventually, the child recovered without neurological sequelae.

## Literature review

We performed a review of the literature written in English and published between 1980 and July 2020. PubMed database, Web of Science Core Collection and Scopus were searched for the published literature by using the terms: “*Campylobacter*” OR “*Campylobacter jejuni*” AND “meningitis” OR “subdural hygroma infection” OR “subdural hematoma infection” AND “children” OR “neonates”. We also searched Google Scholar for other open access resources. We identified and included five articles describing 15 cases of *C. jejuni* CNS infection in children (Table 1). Fourteen cases of neonatal meningitis are described, three sporadic and 11 in a health-care associated outbreak [5, 12–14]. One article described the occurrence of a subdural hygroma infection in 2.5 year old child [15]. The median age of neonates with *C. jejuni* meningitis was 10.6 days (range 5 to 16 days) [5, 12–14]. Only the patient with subdural space infection had underlying brain abnormality; it was a consequence of Sturge-Weber syndrome, with VP shunt inserted to relieve hydrocephalus [15]. Regarding the antimicrobial therapy—the patient with subdural space infection was treated with intravenous chloramphenicol and gentamicin subdural instillation. A subdural-peritoneal shunt was inserted two weeks after the completion of antimicrobial therapy [15]. Patient with meningitis described in 1980 was treated with chloramphenicol and gentamicin for 7 days [12]. Neonates from the nosocomial outbreak were treated with a combination of ampicillin and gentamycin for 7 days, followed by amoxicillin by mouth for 2–3 weeks [5]. Two patients were treated with cefotaxime for 3 weeks [13, 14]. Treatment led to full recovery in all patients except in one who developed moderate dilation of the left ventricle on follow-up [5, 12–15].

**Table 1:** Characteristics of 15 pediatric patients with *C. jejuni* CNS infection

Case	Age/sex	Sample	Identification method	Clinical features	Treatment	References
1	16 days/male	CSF	MALDI-TOF	Meningitis	Ampicillin plus cefotaxime, followed by cefotaxime 2 weeks	Guerrero García et al. [14]
2	15 days/male	CSF	Culture	Meningitis	Cefotaxime 3 weeks	Tsoni et al. [11]
3	30 months/female	CSF from subdural space collection	Culture	Subdural space collection infection	Chloramphenicol along with subdural instillation of gentamycin for 4 weeks	Richie et al. [15]
4	13 days/female	No isolate	No isolate	Meningitis	Ampicillin plus gentamycin 7 days, followed by amoxicillin 2–3 weeks	Goossens et al. [5]
5	11 days/female	No isolate	No isolate	Meningitis	Ampicillin plus gentamycin 7 days, followed by amoxicillin 2–3 weeks	Goossens et al. [5]
6	8 days/male	Blood	Culture	Meningitis	Ampicillin plus gentamycin 7 days, followed by amoxicillin 2–3 weeks	Goossens et al. [5]
7	16 days/female	No isolate	No isolate	Meningitis	Ampicillin plus gentamycin 7 days, followed by amoxicillin 2–3 weeks	Goossens et al. [5]
8	15 days/male	No isolate	No isolate	Meningitis	Ampicillin plus gentamycin 7 days, followed by amoxicillin 2–3 weeks	Goossens et al. [5]
9	5 days/female	Blood	Culture	Meningitis	Ampicillin plus gentamycin 7 days, followed by amoxicillin 2–3 weeks	Goossens et al. [5]
10	14 days/male	No isolate	No isolate	Meningitis	Ampicillin plus gentamycin 7 days, followed by amoxicillin 2–3 weeks	Goossens et al. [5]
11	5 days/female	Blood	Culture	Meningitis	Ampicillin plus gentamycin 7 days, followed by amoxicillin 2–3 weeks	Goossens et al. [5]
12	7 days/male	Blood	Culture	Meningitis	Ampicillin plus gentamycin 7 days, followed by amoxicillin 2–3 weeks	Goossens et al. [5]
13	7 days/female	CSF, blood, stool	Culture	Meningitis	Ampicillin plus gentamycin 7 days, followed by amoxicillin 2–3 weeks	Goossens et al. [5]
14	5 days/female	CSF, stool	Culture	Meningitis	Ampicillin plus gentamycin 7 days, followed by amoxicillin 2–3 weeks	Goossens et al. [5]
15	12 days/male	CSF	Culture	Meningitis	Chloramphenicol plus gentamycin 7 days	Thomas et al. [12]

*CSF* cerebrospinal fluid, *MALDI-TOF* matrix assisted laser desorption/ionization mass spectrometry with time-of-flight detector

## Discussion and conclusion

Infection with *Campylobacter* spp. most often presents as an acute, self-limited gastrointestinal disease. Extraintestinal manifestations of *Campylobacter* infection have been described in rare cases, and include meningitis, endocarditis, septic arthritis, osteomyelitis, and neonatal sepsis [7].

Only one case of subdural space infection caused by *C. jejuni* has been described before—in a child with underlying brain abnormality and VP shunt. The child presented with anorexia, lethargy and vomiting, and *C. jejuni* was cultivated from subdural fluid sample [15]. Other reported *C. jejuni* CNS infections were neonatal meningitis [5, 12–14]. Clinical presentations were in the majority of patients described as nonspecific, such as convulsions, somnolence and lethargy [5, 14, 15]. Fever was the sole symptom in two neonates [12, 13]. None of the neonates with *C. jejuni* meningitis had other risk factors. As was described in our study, infection of fluid collection in the subdural space can present as fever of unknown origin (FUO) and produce no neurological symptoms. Prolonged fever with a history of prior head trauma in adults and children should raise suspicion of intracranial infection.

In the reviewed cases, *C. jejuni* was isolated from the CSF in three patients and in blood culture in four [5, 12, 13, 15]. In one patient *C. jejuni* was detected in CSF using matrix assisted laser desorption/ionization mass spectrometry with time-of-flight detector (MALDI-TOF MS) [14]. This is the first case report of pediatric CNS *Campylobacter* infection being diagnosed due to use of 16sDNA sequence analysis. Molecular detection of *Campylobacter* spp. by 16S rDNA PCR resulted with prompt reduction of empirical antimicrobial therapy and VP shunt removal. This method is rapid and non-specific, and can be especially useful for pathogens that are biochemically inert, fastidious, neglected or slow-growing [8, 16]. Drancourt et al. proposed guidelines for including 16S rDNA sequencing as a reverence method for bacterial identification, especially for slow-growing and fastidious organisms which can remain unidentifiable after the application of all available phenotypic tests [8]. For the genus *Campylobacter*, 16S DNA sequencing does however lack the sufficient discriminatory power to distinguish *C. jejuni* and *C. coli* so the final species identification was performed by hippurate hydrolysis test of the achieved isolate.

World Health Organization does not recommend chloramphenicol treatment for bacterial meningitis as the first-line therapy due to its toxicity and carcinogenic potential in humans [17]. High rates of resistance make cephalosporins poor choice for *C. jejuni* meningitis [7]. Although *C. jejuni* is susceptible to gentamicin, its nephrotoxicity and ototoxicity, as well as poor CNS penetration, make it an inappropriate choice for meningitis treatment [18–20]. Fluoroquinolones and tetracyclines have demonstrated favorable CNS penetration in adults, however data are limited due to their potential pediatric-specific toxicities [20–23]. Imipenem and meropenem are the only carbapenems with pediatric data. As imipenem is related to higher seizure risk, meropenem is considered to be a safer option in CNS infection management [20, 24]. Good CNS penetration, low rate of adverse reactions, and good bacterial susceptibility were in favor of meropenem as the best choice in our patient [20]. There are no high-quality evidence guidelines regarding the duration of treatment of bacterial meningitis. Recommended duration for uncomplicated meningitis depends on the causative agent. Three weeks of therapy or a minimum of two weeks beyond the first sterile CSF culture is recommended for gram-negative meningitis, whatever of above is longer [25]. There are only several case reports describing two to three weeks meropenem treatment of *C. jejuni* meningitis in adults [26–28]. We decided for 4-weeks meropenem treatment. Initial treatment failure in our patient could be explained by the size of the hygroma and development of chronic subdural infection. In this setting, the neurosurgical evacuation was necessary for successful treatment.

In conclusion, we report a case of *C. jejuni* subdural hygroma infection in a patient with holoprosencephaly and VP shunt. Although we didn’t cultivate *Campylobacter* from stool samples, parents reported regular poultry consumption. Therefore, bacteremia from gastrointestinal tract probably resulted in infection of the posttraumatic subdural hygroma. Congenital brain abnormalities together with the brain injury probably made our patient more prone to *C. jejuni* CNS invasion. Since *C. jejuni* meningitis is most commonly diagnosed in neonates, this pathogen is often neglected as a causative agent for CNS infection in older children. Regardless of unspecific clinical presentation, sudden onset of symptoms along with the history of prior closed head trauma, and exclusion of VP shunt meningitis should always raise suspicion of subdural space infection. Early adequate treatment of CNS infections is crucial for satisfying recovery and prompt detection of a pathogen has a significant role in the choice of antimicrobial therapy. Therefore, rapid broad-range molecular methods represent an irreplaceable tool for the detection of rare or unexpected pathogens. Localized subdural infection may require a prolonged antibiotic therapy. In cases of subdural space infection surgical treatment should be considered. In our case, 4-weeks of meropenem treatment combined with surgical evacuation lead to a good clinical outcome.

## Data Availability

The datasets generated and/or analysed during the current study are available in the GenBank repository, number OP090654, https://www.ncbi.nlm.nih.gov/nuccore/OP090654.

## Abbreviations

BLASTn: Nucleotide basic local alignment search tool
CNS: Central nervous system
CRP: C-reactive protein
CSF: Cerebrospinal fluid
CT: Computer tomography
EVD: External ventricular drainage system
FUO: Fever of unknown origin
MALDI-TOF MS: Matrix assisted laser desorption/ionization mass spectrometry with time-of-flight detector
MIC: Minimal inhibitory concentration
MRI: Magnetic resonance imaging
SED: Subdural external drainage system
VP: Ventriculoperitoneal
